# Prospects for Fungal Bioremediation of Acidic Radioactive Waste Sites: Characterization and Genome Sequence of *Rhodotorula taiwanensis* MD1149

**DOI:** 10.3389/fmicb.2017.02528

**Published:** 2018-01-08

**Authors:** Rok Tkavc, Vera Y. Matrosova, Olga E. Grichenko, Cene Gostinčar, Robert P. Volpe, Polina Klimenkova, Elena K. Gaidamakova, Carol E. Zhou, Benjamin J. Stewart, Mathew G. Lyman, Stephanie A. Malfatti, Bonnee Rubinfeld, Melanie Courtot, Jatinder Singh, Clifton L. Dalgard, Theron Hamilton, Kenneth G. Frey, Nina Gunde-Cimerman, Lawrence Dugan, Michael J. Daly

**Affiliations:** ^1^Department of Pathology, Uniformed Services University of the Health Sciences, Bethesda, MD, United States; ^2^Henry M. Jackson Foundation for the Advancement of Military Medicine, Bethesda, MD, United States; ^3^Department of Biology, Biotechnical Faculty, University of Ljubljana, Ljubljana, Slovenia; ^4^Lawrence Livermore National Laboratory, Computing Applications and Research Department, Livermore, CA, United States; ^5^Biosciences and Biotechnology Division, Physics and Life Sciences Directorate, Lawrence Livermore National Laboratory, Livermore, CA, United States; ^6^European Molecular Biology Laboratory, European Bioinformatics Institute, Cambridge, United Kingdom; ^7^Collaborative Health Initiative Research Program, Uniformed Services University of the Health Sciences, Bethesda, MD, United States; ^8^Department of Anatomy, Physiology and Genetics, Uniformed Services University of the Health Sciences, Bethesda, MD, United States; ^9^The American Genome Center, Bethesda, MD, United States; ^10^Biological Defense Research Directorate, Naval Medical Research Center, Fredrick, MD, United States

**Keywords:** bioremediation, yeasts, radiation resistance, heavy metal resistance, pH minimum, temperature maximum, *Rhodotorula taiwanensis*, genome

## Abstract

Highly concentrated radionuclide waste produced during the Cold War era is stored at US Department of Energy (DOE) production sites. This radioactive waste was often highly acidic and mixed with heavy metals, and has been leaking into the environment since the 1950s. Because of the danger and expense of cleanup of such radioactive sites by physicochemical processes, *in situ* bioremediation methods are being developed for cleanup of contaminated ground and groundwater. To date, the most developed microbial treatment proposed for high-level radioactive sites employs the radiation-resistant bacterium *Deinococcus radiodurans*. However, the use of *Deinococcus* spp. and other bacteria is limited by their sensitivity to low pH. We report the characterization of 27 diverse environmental yeasts for their resistance to ionizing radiation (chronic and acute), heavy metals, pH minima, temperature maxima and optima, and their ability to form biofilms. Remarkably, many yeasts are extremely resistant to ionizing radiation and heavy metals. They also excrete carboxylic acids and are exceptionally tolerant to low pH. A special focus is placed on *Rhodotorula taiwanensis* MD1149, which was the most resistant to acid and gamma radiation. MD1149 is capable of growing under 66 Gy/h at pH 2.3 and in the presence of high concentrations of mercury and chromium compounds, and forming biofilms under high-level chronic radiation and low pH. We present the whole genome sequence and annotation of *R. taiwanensis* strain MD1149, with a comparison to other *Rhodotorula* species. This survey elevates yeasts to the frontier of biology's most radiation-resistant representatives, presenting a strong rationale for a role of fungi in bioremediation of acidic radioactive waste sites.

## Introduction

Between 1945 and 1986, immense volumes of radioactive waste were generated from the production of 46,000 nuclear weapons in the United States. This was a period of history when national security priorities often surmounted concerns over the environment. Many Cold War wastes contained mixtures of inorganic contaminants including radionuclides (e.g., U and Tc), heavy metals (e.g., Cr and Hg), and nitrate, which were disposed directly to the ground at 120 sites across the United States (Daly, 2000). As the processing of uranium ores involved dissolution and extraction with nitric acid, this led to large volumes of highly acidic radioactive waste, which were stored in subterranean holding tanks or ponds. Over the past six decades, low levels of widespread contamination originating from such waste sites have contaminated over 7.0 × 10^7^ m^3^ of surface and subsurface soils, and over 3.0 × 10^12^ L of groundwater (McCullough et al., 1999; Daly, 2000). As a result of the chemical reprocessing of 1.1 × 10^8^ kg of nuclear fuel at the Hanford Site (WA, USA) alone, 2.1 × 10^5^ m^3^ of radioactive waste were produced at nine reactors and stored in 177 underground tanks. These storage tanks with a lifespan of 10–20 years have been used since 1943, and the first leaks were confirmed in 1959. The amount of waste leakage from the Hanford tanks continues to grow, with estimates in 2004 ranging from 2.3 to 3.7 × 10^6^ L (Fredrickson et al., 2004). The scale of these waste environments leaves few options for cleanup other than bioremediation (Brim et al., 2000).

In 2000, more than 110 distinct aerobic heterotrophic bacteria were isolated from below Hanford tank SX-108, which has been leaking extremely radioactive waste since the 1960s (Fredrickson et al., 2004). Among the numerous bacteria identified, *Arthrobacter* spp. were the most prevalent and *Deinococcus* spp. the most radiation-resistant. Both bacterial genera are known for their ability to survive harsh environmental conditions and reduce a variety of metals, and for their dependence on Mn for growth and resistance (Daly et al., 2004; Fredrickson et al., 2004; Ehrlich and Newman, 2008). The isolation of *Deinococcus radiodurans* from sediments under tank SX-108 focused research on this extremophile: first, to engineer metal-reducing and organic toxin-degrading capabilities into this bacterium; and second, to test the ability of engineered *D. radiodurans* to reduce/immobilize different metals, and to couple those reactions to solvent degradation while growing under high-level chronic ionizing radiation (CIR). Metal reduction coupled to toluene degradation as a bioremediation strategy for radioactive sites was successfully demonstrated in *D. radiodurans* at near-neutral pH under CIR (60 Gy/h) (Brim et al., 2006). However, *D. radiodurans* strain R1 and its engineered counterparts cannot grow at pH values below 4.8 (unpublished results).

To determine whether or not radiation-resistant acidophilic microorganisms exist, we first screened approximately 60 different environmental samples (desert sands, acid mine drainages, soils) for microorganisms that are able to grow under 36 Gy/h at pH 2.3. This yielded the basidiomycetous yeast *Rhodotorula taiwanensis* MD1149, which can grow under 66 Gy/h at pH 2.3. Fungi play an important role in the biogeochemical cycling of manganese and other redox-active metals (Ehrlich and Newman, 2008; Culotta and Daly, 2013), which is related to their ability to survive radiation and other oxidative challenges (Gadd, 2007; Daly, 2009; Sharma et al., 2017). Nevertheless, any prospect of yeasts in bioremediation of radioactive waste sites has been neglected, mainly due to the lack of research in this nascent field of radiomycology; preliminary fungal isolates from beneath tank SX-108 were dismissed as contaminants (Fredrickson et al., 2004). We therefore screened 26 additional yeasts of the Microbial Culture Collection EX^1^. These EX yeast strains (EXF) and MD1149 were tested for their resistance to ionizing radiation (chronic and acute), heavy metal resistance, their pH minima and temperature maxima, and for their ability to form biofilms. From among the numerous CIR- and heavy metal-resistant yeasts identified, we judged MD1149 as the most suitable for bioremediation of acidic radioactive sites, therefore justifying its whole genome sequencing. We present a comparative analysis of MD1149 with three other *Rhodotorula* spp. Our analysis of the core metabolic and stress-resistance characteristics of MD1149, together with the identification of several yeasts capable of growth at low pH under high-level chronic γ-irradiation, strengthens the rationale for the important role of fungi in bioremediation of radioactive Cold War environmental waste sites.

## Materials and methods

### Radiological, chemical, and biological safety

All experimental work was performed under standard laboratory safety conditions, and all radiological, chemical, and biological safety precautions were observed following rules and regulations established for respective research institutions.

### Strains, isolation of MD1149, and irradiations

The ascomycetous and basidiomycetous yeasts used in this study and their isolation sites are presented in Table 1.

**Table 1:** Ranking of representative fungi by the survival index D_10_ together with other characteristics.

**Strain #**	**Name**	**Phylum**	**Isolated from**	**D_10_ [kGy]**	**T_max_ [°C]**	**T_opt_ [°C]**	**AM, pH 2.3, CIR**	**YPD, pH 7, CIR**	**AN, YPD**	**pH_min_ AM**	**pH_min_ YPD**	**AM, HgCl_2_ [μM]**	**AM, MER [μM]**	**AM, CrCl_3_ [μM]**	**AM, K_2_Cr_2_O_7_ [μM]**
EXF-5294	*Saccharomyces cerevisiae*	Ascomycota	Red wine, Slovenia	3.2	40	30	–	–	+	3.0	2.5	25	<500	>500	>10
EXF-6408	*Metschnikowia fructicola*	Ascomycota	Mofette, CO_2_ rich water, Slovenia	3.0	<40	23.5	+	+	w	2.5	2.5	10	1,000	500	100
EXF-4909	*Saccharomyces bayanus* x *cerevisiae*	Ascomycota	New wine, Slovenia	3.0	40	26	+	+	+	2.5	2.5	25	<1,000	500	>500
EXF-5281	*Saccharomyces* cerevisiae	Ascomycota	Floor spilled with wine, Slovenia	2.6	40	30	+	+	–	2.5	2.5	10	>50	>100	>250
MD1149	*Rhodotorula taiwanensis*	Basidiomycota	Acid mine drainage, USA	2.5	32	25	+	+	–	1.5	2	50	>500	500	300
EXF-6398	*Pichia kudriavzevii*	Ascomycota	Mofette, Slovenia	2.0	45	27	+	+	+	1.5	2.0	25	100	500	750
EXF-308	*Rhodotorula rubra*	Basidiomycota	Glacial ice in sea water, Svalbard	2.0	37	30	+	+	–	2.0	2.0	25	1,000	750	100
EXF-5293	*Saccharomyces bayanus*	Ascomycota	Apple juice, Slovenia	2.0	40	26	+	+	+	2.5	2.5	25	3,000	>500	>100
EXF-6464	*Debaryomyces hansenii*	Ascomycota	Water from slow moving creek showing CO_2_ release	1.8	<40	26	+	+	–	2.0	2.0	10	100	500	250
EXF-7288	*Saccharomyces kudriavzevii*	Ascomycota	Bark of *Quercus ilex*, Croatia	1.5	<40	24	–	–	–	2.5	2.5	10	>50	>100	>250
EXF-3501	*Rhodosporidium diobovatum*	Basidiomycota	Ice, Svalbard	1.4	37	24	+	+	–	2.0	2.0	25	1,000	750	100
EXF-6402	*Kazachstania exigua*	Ascomycota	Mofette, CO_2_ rich water, Slovenia	1.2	<40	27	+	+	+	2.0	2.0	10	100	750	50
EXF-3697	*Rhodosporidium kratochvilovae*	Basidiomycota	Ice, Svalbard	1.2	37	30	+	+	–	2.5	1.5	50	1,000	500	250
EXF-1534	*Rhodotorula lysinophila*	Basidiomycota	Glacial ice in sea water, Svalbard	1.1	37	25	–	+	–	3.0	3.0	25	1,000	500	250
EXF-1529	*Rhodotorula minuta*	Basidiomycota	Glacial ice in sea water, Svalbard	1.1	37	25	+	+	–	2.5	2.0	25	1,000	500	250
EXF-5557	*Rhodotorula slooffiae*	Basidiomycota	Box of plasticizer in the washing machine, Slovenia	1.1	37	30	+	+	–	2.0	2.0	25	100	500	250
EXF-3409	*Cryptococcus liquefaciens*	Basidiomycota	Glacier ice, Svalbard	1.0	<40	30	+	+	–	2.5	>3	25	1,000	750	50
EXF-6453	*Cyberlindnera saturnus*	Ascomycota	Mofette, soil, Slovenia	1.0	<40	25	+	+	+	2.5	2.0	25	500	2,000	>3,000
EXF-1496	*Pichia guilliermondi*	Ascomycota	Glacial ice, Svalbard	1.0	43	25	+	+	–	2.0	2.0	25	250	750	1,500
EXF-6094	*Rhodotorula calyptogenae*	Basidiomycota	Dishwasher rubber, France	1.0	40	26	+	+	–	2.5	7.0	25	1,000	500	100
EXF-7210	*Saccharomyces kudriavzevii*	Ascomycota	Bark of *Quercus* sp, Montenegro	1.0	<40	24	–	–	+	2.5	2.5	10	>50	>100	>250
EXF-7289	*Saccharomyces kudriavzevii*	Ascomycota	Bark of *Quercus ilex*, Croatia	1.0	<40	24	–	–	+	2.5	2.5	10	>50	>100	>250
EXF-3800	*Rhodotorula benthica*	Basidiomycota	Glacier ice with sediment, Svalbard	0.9	25	25	–	–	–	2.5	2.0	10	1,000	500	75
EXF-3909	*Rhodotorula laryngis*	Basidiomycota	Sea water near the glacier, Svalbard	0.9	25	20	–	–	–	2.5	2.5	25	1,000	750	25
EXF-7964	*Wickerhamomyces anomalus*	Ascomycota	Forest ditch water, Slovenia	0.9	40	25	+	+	+	2.5	2.5	10	1,500	>3,000	1,500
EXF-6463	*Candida pseudolambica*	Ascomycota	Mofette, CO_2_ rich water, Slovenia	0.5	<40	33	+	+	–	2.0	2.5	10	1,500	>3,000	75
EXF-589	*Debaryomyces hansenii*	Ascomycota	By the Atlantic coast, Namibia	0.3	<40	23	–	–	–	>3	>3	10	100	250	100

*Temperature maximum supporting growth (T_max_), ability to grow under CIR (36 Gy/h) on solid AM (pH 2.3) or YPD (pH 7). Growth under anaerobic conditions (AN), pH minimum (pH_min_), and the highest heavy metal concentrations compatible with growth in medium supplemented with HgCl_2_ (Hg^2+^), merbromin (MER), CrCl_3_ (Cr^3+^), and K_2_Cr_2_O_7_ (Cr^6+^); growth (+), no growth (−), weak growth (w)*.

Sixty environmental samples were collected between 2001 and 2015 as a part of a larger study. These samples represent desert sands (Arizona, Nevada, New Mexico); dried plant debris from deserts (Arizona, Nevada, New Mexico); water, sediments, and soil from abandoned mines and mine drainages (coal mine in Maryland, silver and gold mines in Colorado, mercury mine in Idrija, Slovenia); hot springs (Colorado; Radenci, Slovenia); water and sediments from acidic river (Rio Tinto, Spain); and radioactive waste storage tanks (Uniformed Services University of the Health Sciences, Maryland). One gram of each environmental sample was resuspended in 10 ml of MQ purified water and allowed to settle for 30 min. One milliliter of the supernatant was added to 10 ml of the oligotrophic medium AM (complex *Acidiphilium* Medium) (San Martin-Uriz et al., 2014) adjusted to pH 2.3 with HNO_3_, and incubated in a shaker incubator (200 rpm) at 25°C for 4 days. One hundred microliters were then spread on AM plates (pH 2.3) and incubated at 25°C under 36 Gy/h. After 3 days of continuous CIR, the plates were inspected for growth. Single colonies were re-inoculated on fresh AM solid medium.

Throughout this work, CIR exposures specified under 36 Gy/h (~22°C) were performed in a ^137^Cs irradiator (GammaCell 40, J. L. Shepard and Associates). For all other CIR exposures, we used a second adjustable dose rate ^137^Cs irradiator (Mark 1 Model 68 A, J. L. Shepard and Associates), also at ~22°C. Acute exposures were performed in a ^60^Co irradiator (10 kGy/h) (J. L. Shepard and Associates) at 0°C.

### Phenotype characterization

The minimum pH and the highest Hg^2+^, merbromin, Cr^6+^, and Cr^3+^ concentrations supporting growth were determined in liquid AM and Yeast Extract-Peptone-Dextrose (YPD)^2^ medium. The overnight (O/N) cultures pre-grown at optimal temperatures were washed twice in sterile MQ and used to inoculate fresh liquid media adjusted for pH with HNO_3_, and/or supplemented with different concentrations of heavy metals to a final OD_600_ ~0.1. The strains were incubated in a shaker incubator, 200 rpm, at optimal temperatures. After inoculation, the OD_600_ was measured every 24 h for 1 week.

Maximum growth temperature and anaerobic growth were determined by observing colony formation on solid YPD medium incubated at various temperatures (25–65°C; temperature maxima); and for anaerobic growth, at a given strain's optimal temperature, in the presence or absence of atmospheric oxygen for 1 week.

Survival following acute forms of γ-radiation was determined on solid YPD medium by colony forming unit (CFU) assay as described previously (Daly et al., 2004). The ability of cells to grow under CIR on YPD pH 7.0 and AM pH 2.3 was monitored visually. The ability of a strain to form biofilms was tested in 96-well microtiter plates, as described by others (O'Toole, 2011), with eight replicate wells for every strain and each condition, and eight wells for blank controls. Pulsed-field gel electrophoresis (PFGE) with MD1149 genomic DNA was performed as described previously (Saracli et al., 2003).

### Organic acid production by MD1149

The OD_600_ of an O/N culture of MD1149 in the Yeast Mold Broth (YM, Difco) was adjusted to 0.05 in modified Hommel's Minimal Salts (HMS; 0.3% (NH_4_)_2_SO_4_, 0.05% NaCl, 0.07% MgSO_4_, 0.04% Ca(NO_3_)_2_, 0.04% K_2_HPO_4_, 0.25% KH_2_PO_4_, 0.06% yeast extract, 5% glucose). At the indicated time (2, 4, 6, and 8 days), OD_600_ was assessed, and 10 ml of culture were centrifuged twice at 5,000 × g to obtain spent liquid medium (SLM).

Organic acids in SLM were identified and measured using a Waters Xevo G2-XS QTOF mass spectrometer (Waters Corporation, Milford, MA USA) coupled with a Waters Acquity H Class chromatography system. Organic acids were separated on a Waters Acquity UPLC HSS C18 1.8 μm 2.1 × 100 mm column using a modification of a previously published method (Fernández-Fernández et al., 2010). Mobile phases were methanol (solvent A) and water with 0.5% formic acid (solvent B). The separation method was as follows: initial, 90% B; 0.1 min, 90% B; 6 min, 70% B; 6.1 min, 90% B; 12 min, 90% B. The flow rate was 125 μl/min. The column compartment thermostat was set at 35°C, and the autosampler tray temperature was maintained at 4°C. Detection was accomplished by mass spectrometry with the electrospray ion source operating in negative ion, resolution mode. Data acquisition was performed using MassLynx Version 4.1 data acquisition software (Waters Corp.), with MS^e^ data-independent centroid acquisition and leucine enkephalin lockmass correction.

Liquid chromatography mass spectrometry (LC-MS) and liquid chromatography tandem-mass spectrometry (LC-MS/MS) settings were as follows: Low Energy, 50–1000 Da, 6 V collision energy; High Energy, 50–1000 Da, collision energy ramp from 10–40 V; Scan time: 0.5 s; Source: 120°C; Desolvation, 450°C; Desolvation gas flow: 800 l/h; capillary voltage: 1.90 kV; Sampling cone voltage: 40 V. Experimental samples were compared with authentic standards (Sigma-Aldrich, St. Louis, MO) to identify organic acids present in cell culture media. Quantitation was performed with Waters TargetLynx software with quadratic calibration curve fitting.

### MD1149 identification, DNA isolation, and genome analysis

MD1149 was first identified at the genus level based on micro- and macro morphology and assimilation test (YT MicroPlate™, BIOLOG Inc.), and then to the species level using genetic molecular identification (Mohamed et al., 2014).

Total DNA was isolated from MD1149 using the Wizard Genomic DNA Purification Kit (Promega, Madison, WI, USA) and quantified by NanoDrop 2000 (Thermo Fisher Scientific).

The ITS1-5.8S rDNA-ITS2 and 18S rDNA sequences were matched to the GenBank non-redundant nucleotide database with the BLASTN algorithm (Altschul et al., 1990). MD1149 and related sequences were analyzed for similarity within the Geneious software package (Kearse et al., 2012) by using MUSCLE alignment (Edgar, 2004). The aligned sequences of representative strains were used to construct a phylogenetic tree with the PhyML 3.0 software (Guindon et al., 2010) with approximate likelihood-ratio test for branch supports, and with six substitution rate categories. The substitution model, alpha parameter of the gamma distribution and the proportion of invariable sites, was estimated by jModelTest 2.0 (Darriba et al., 2012).

The draft genome was generated using a combination of Illumina and 454 technologies. Two short-insert paired-end libraries, a fragment, 625-bp insert size (2 × 300 bp reads) and an overlapping fragment, 405-bp insert size (2 × 300 bp reads) were sequenced using version 3 chemistry on the MiSeq (Illumina, Inc., San Diego, CA, USA) (Bennett, 2004). Two large-insert paired-end libraries (8-kbp and 20-kbp insert size) were constructed and sequenced on the 454 GS FLX (Roche/454 Life Sciences, Branford, CT, USA) (Margulies et al., 2005). The draft data was assembled *de novo* with CLC Genomics Workbench v9.0 (QIAGEN Aarhus, Denmark). Repetitive sequences were identified using RepeatMasker (Smit et al., 2013–2015) and RepBase library (Jurka et al., 2005). The genome assembly completeness was evaluated with the Benchmarking Universal Single-Copy Orthologs (BUSCO 1.22) (Simão et al., 2015) software using the dataset for fungi.

For pairwise genome alignments, the following genomes were used: *Rhodotorula* sp. (Goordial et al., 2016), *R. mucilaginosa* (Deligios et al., 2015), *R. glutinis* (Paul et al., 2014), *R. toruloides* (Zhang et al., 2016), *R. graminis* (Firrincieli et al., 2015), *Puccinia graminis* (Duplessis et al., 2011). The genome alignments of contigs longer than 100 kbp were calculated with the PROmer algorithm, as implemented in MUMmer 3.23, and plotted with the MUMmerplot utility (Kurtz et al., 2004) as described by Hane et al. (2011).

### RNA isolation and genome annotation

MD1149 total RNA was isolated, then pooled and sequenced from cells grown under the following conditions: YPD (25°C, O/N and 3 days), YNB + 2% glucose (25°C, O/N and 3 days), YNB + 2% glucose (6°C and 37°C, O/N), YNB + 2% glycerol (25°C, O/N), AM pH 2.3 (25°C, O/N, 0 and 36 Gy/h), and AM pH 7 (25°C, O/N, 0 and 36 Gy/h). The RNA was isolated from MD1149 using RiboPure RNA Purification Kit for Yeasts (Thermo Fisher Scientific). RNA integrity was assessed by Fragment Analyzer (Advanced Analytical Technologies Inc., Ankeny, IA, USA). Sequencing libraries were prepared from 500 ng of total RNA input using the TruSeq Stranded mRNA Library Preparation Kit (Illumina) with barcoded adapters. Sequencing libraries yield and concentration were determined using the Illumina/Universal Library Quantification Kit (KAPA Biosystems, Wilmington, MA, USA) on the Light Cycler 480 (Roche Diagnostics Co., Indianapolis, IN, USA). Library size distribution was determined using the Fragment Analyzer™ (Advanced Analytical Technologies Inc.). Clustering and sequencing was performed on the NextSeq 500 (Illumina) with paired-end reads of 75 bp length.

RNAseq reads were quality trimmed with Sickle (Joshi and Fass, 2011) and aligned to the assembled genome with TopHat 2.1.1 (Kim et al., 2013). The alignment was then used for the transcriptome assembly with Trinity 2.2.0 (Haas et al., 2013) in Genome Guided mode with jaccard clipping and a maximum intron length of 1,500 bp. Protein-coding and tRNA genes were annotated using MAKER 2.31.8 (Campbell et al., 2014). The complete Swissprot database was used as evidence, along with the database of BUSCO, a set of Basidiomycete fungal proteomes and the sequenced transcriptome of MD1149. Three gene predictors were used in the MAKER pipeline: SNAP (Korf, 2004; Campbell et al., 2014), GeneMark-ET (Lomsadze et al., 2014), and Augustus (Stanke and Waack, 2003). This Whole Genome Shotgun project has been deposited at DDBJ/ENA/GenBank under the accession PJQD00000000. The version described in this paper is version PJQD01000000.

Predicted protein sequences from the MD1149 genome were processed through automated functional annotation using the PSAT metaserver (Leung et al., 2016), which ran EFICAz 2.5 (Kumar and Skolnick, 2012), blastp against the KEGG, MetaCyc, BRENDA, and STRING databases (Caspi et al., 2014; Chang et al., 2015; Szklarczyk et al., 2015; Kanehisa et al., 2016), and Interproscan (Jones et al., 2014). Additionally, protein sequences were processed through online servers running SignalP 4.0 (Petersen et al., 2011) and TMHMM 2.0 (Krogh et al., 2001). The pepstats utility (EMBOSS suite) was used to calculate protein properties (Rice et al., 2000).

GO annotations were also quantified and then projected into the full GO hierarchy using slim-o-matic (Courtot et al., 2016) and reviewed using Protege (Munsen, 2015). High-level categories were selected, into which the MD1149 GO annotations were mapped (slimmed). Categories corresponding to GO molecular function and biological process were quantified and expressed as percent of annotations across the MD1149 genome.

Predicted proteins from MD1149, *R. graminis, R*. sp. JG-1b, and *R. toruloides* were compared by all-against-all blastp at identity cutoff 95% and query coverage ≥95% using CGP^3^, and post-processed to identify fasta sequences unique or in common among the species and putatively duplicated within each species. Sequence logos of conserved nucleotide positions in all introns of median length were drawn using WebLogo 3 (Crooks et al., 2004).

## Results

### Isolation of MD1149

**Figure 1:**
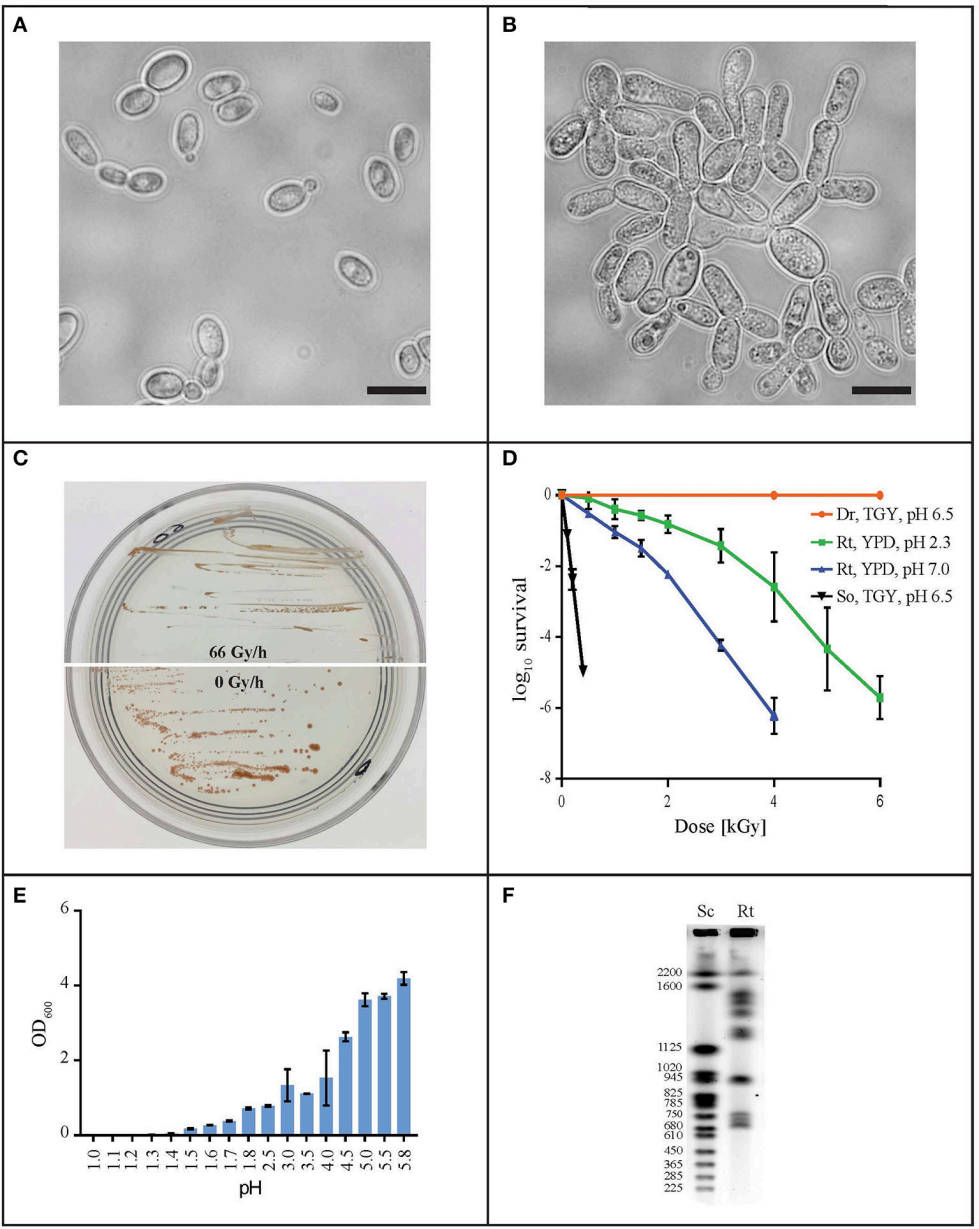
Characterization of *R. taiwanensis* MD1149. Light microscopy of liquid O/N culture of MD1149 grown **(A)** without CIR and **(B)** under 36 Gy/h (^137^Cs). Scale bars: 5 μm. **(C)** Growth of MD1149 on AM plates at pH 2.3 under 66 Gy/h, and without CIR. **(D)** Survival after acute gamma irradiation (^60^Co) of MD1149 (Rt) pre-grown in and recovered on YPD at pH 2.3 and 7.0. Model bacteria *D. radiodurans* (Dr) and *Shewanella oneidensis* (So) were pre-grown in and recovered on TGY, pH 6.5. **(E)** pH-dependent growth of MD1149 in YPD (pH adjusted with HNO_3_) after 48 h. **(F)** Chromosomal partitioning in MD1149 by PFGE (Rt) and in *S. cerevisiae* size ladder (Sc).

**Figure 2:**
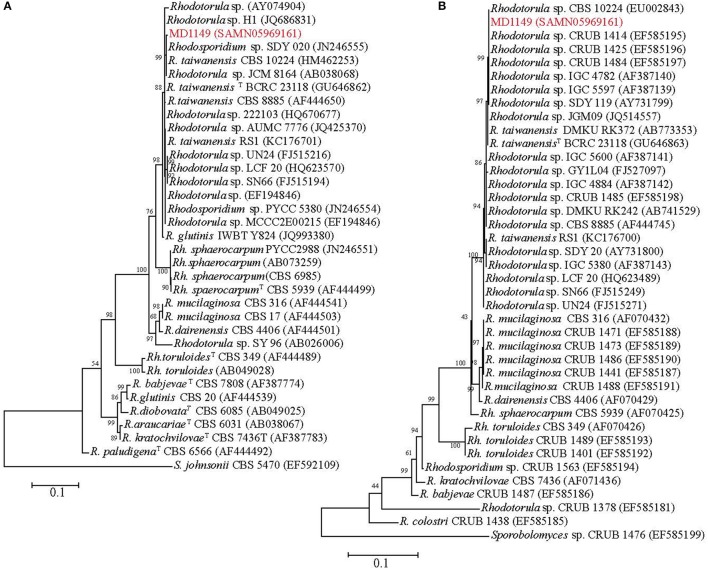
Phylogenetic analysis of *R. taiwanensis* MD1149 and related strains, and *Sporobolomyces* spp. as the root. The phylogenetic tree was constructed based on **(A)** ITS1-5.8S rDNA-ITS2 and **(B)** 18S rDNA sequences. GenBank accession number of each strain's sequence is in parentheses. ^T^Type strain.

In order to find a suitable candidate for bioremediation of acidic radioactive environmental waste sites, we first screened a variety of aquatic and terrestrial environments (desert sands, acid mine drainages, soils, and water samples) for strains that are both acid- and CIR-resistant. This strategy yielded only one strain, named MD1149, isolated from a sediment sample from an abandoned acid mine drainage facility in Maryland, USA (39°31′34.22″N, 79°1′12.16″W). MD1149 is a red-pigmented, unicellular, non-sporulating, ovoidal, obligately aerobic, budding yeast (Figure 1A, Table 1), which became pleomorphic under 36 Gy/h (Figure 1B). Phylogenetic analysis based on the internal transcribed spacer (ITS) and small subunit rRNA (SSU) sequences identified MD1149 as the basidiomycetous yeast *R. taiwanensis* [closely related to the type strain BCRC 23118(T) = CBS 11729(T)] (Figure 2), and confirmed by its micromorphological, macromorphological, and physiological characteristics (data not shown). Based on PFGE, the genome size of MD1149 was estimated to be >13 Mbp (Figure 1F). MD1149 was deposited with the Microbial Culture Collection EX as EXF-12971. Since other environmental samples screened did not yield additional acid- and CIR-resistant strains, we extended our study by including 26 distinct yeasts from the Microbial Culture Collection EX (Table 1).

### Radiation resistance

Like most of the tested yeasts in this study, MD1149 was capable of growing luxuriantly at pH 2.3 and 7.0 under 36 Gy/h (Table 1). However, it was the only strain capable of growth under 66 Gy/h (Figure 1C).

Survival assays yield a radiation resistance metric named D_10_, which represents the acute radiation dose (Gy) giving 10% CFU survival (Daly et al., 2007, 2010; Sharma et al., 2017). Among tested strains, the most resistant was *Saccharomyces cerevisiae* EXF-5294 (D_10_, 3.2 kGy), and the most sensitive was *Debaryomyces hansenii* (D_10_, 0.3 kGy). The D_10_ of MD1149 is 2.5 kGy and ranks among the most radiation-resistant yeasts identified both for acute and chronic exposures (Table 1). Importantly, the radiation resistance of MD1149 increased with decreasing pH, from D_10_ 0.8 kGy at pH 7.0 to D_10_ 2.5 kGy at pH 2.3 (Figure 1D).

### Temperature optima and maxima

Optimal and maximum growth temperatures for the strains are reported in Table 1. *Pichia kudriavzevii* was the most thermotolerant and could grow at 45°C. The temperature maximum of MD1149, which optimally grows at 20–25°C, was 32°C. The most temperature-sensitive strains were *R. benthica* and *R. larynges*, which could grow at 25°C or below.

### pH minima

Over the course of 7 days, growth of strains in YPD and AM media adjusted to different pH values was montiored spectrophotometrically. Growth was considered as increasing when the OD_600_ rose above 0.1. The pH minima for growth of the yeasts are presented in Table 1. A full pH-dependent growth response curve for MD1149 is presented (Figure 1E). As shown in Table 1, the pH minima supporting growth of the yeast in rich (YPD) or oligotrophic (AM) media were very similar. *Rhodotorula calyptogenae* was the only strain that could not grow in YPD at low pH, whereas it grew well in AM at pH 2.5. Remarkably, growth responses of MD1149 and *Pichia kudriavzevii* in AM and *Rhodosporidium kratochvilovae* in YPD showed that their pH-minima approximate 1.5 (Figure 1E, Table 1).

### Heavy metal resistance

**Figure 3:**
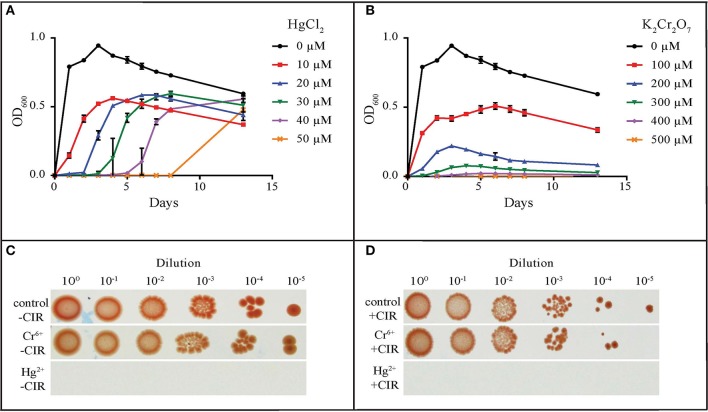
Resistance of *R. taiwanensis* MD1149 to HgCl_2_ and K_2_Cr_2_O_7_. Growth in liquid AM supplemented with **(A)** HgCl_2_ and **(B)** K_2_Cr_2_O_7_. **(C)** Growth of diluted cell suspension (OD_600_ ~0.9) on solid AM with no metals added (control), with 100 μM K_2_Cr_2_O_7_ (Cr^6+^), and with 30 μM HgCl_2_ (Hg^2+^), no CIR. **(D)** As for Panel **(C)**, under 36 Gy/h (+CIR). For corresponding CrCl_3_ (Cr^3+^) results, see Table 1.

The most common metal contaminants at DOE sites are U, Sr, Cs, Tc, Cr, Pb, and Hg. Among these, U, Tc, Hg, and Cr are significantly less mobile when reduced, and are capable of being immobilized by microorganisms (Daly, 2000). We tested yeasts for their resistance to Hg and Cr: mercury in the form of HgCl_2_ (Hg^2+^) and merbromin (organo-Hg), and chromium in the form of CrCl_3_ (Cr^3+^) and K_2_Cr_2_O_7_ (Cr^6+^). Table 1 summarizes heavy metal tolerances for strains grown in oligotrophic medium (AM) instead of YPD; YPD contains phosphates and myriad small organic molecules (e.g., peptides) that can mask metal toxicity (e.g., Mergeay, 1995). As expected, Hg^2+^ in HgCl_2_ is considerably more toxic than Cr^3+^ and Cr^6+^. The two strains most resistant to Hg^2+^, Cr^3+^, and Cr^6+^, were MD1149 and *R. kratochvilovae* (Figure 3 and Table 1), which could grow in AM supplemented with 50 μM HgCl_2_, and at significantly higher concentrations of Cr^3+^ and Cr^6+^. In contrast, most strains were resistant to millimolar concentrations of Hg when added as merbromin. *Wickerhamomyces anomalus* and *Candida pseudolambica* could grow in liquid medium supplemented with 3 mM Cr^3+^, whereas MD 1149 was resistant only to 0.5 mM Cr^3+^ (Table 1). The growth responses of MD1149 in AM supplemented with increasing concentrations of HgCl_2_ or K_2_Cr_2_O_7_ were distinct. Whereas increasing the concentration of Hg^2+^ increased the length of the lag-phase before the onset of exponential growth, increasing the concentration of Cr^6+^ slowed the growth of MD1149 (Figures 3A,B). Furthermore, in contrast to Cr^6+^/Cr^3+^, we showed that Hg^2+^ had a significant detrimental effect on MD1149 growth under CIR or not (Figures 3C,D).

### Biofilm formation

**Figure 4:**
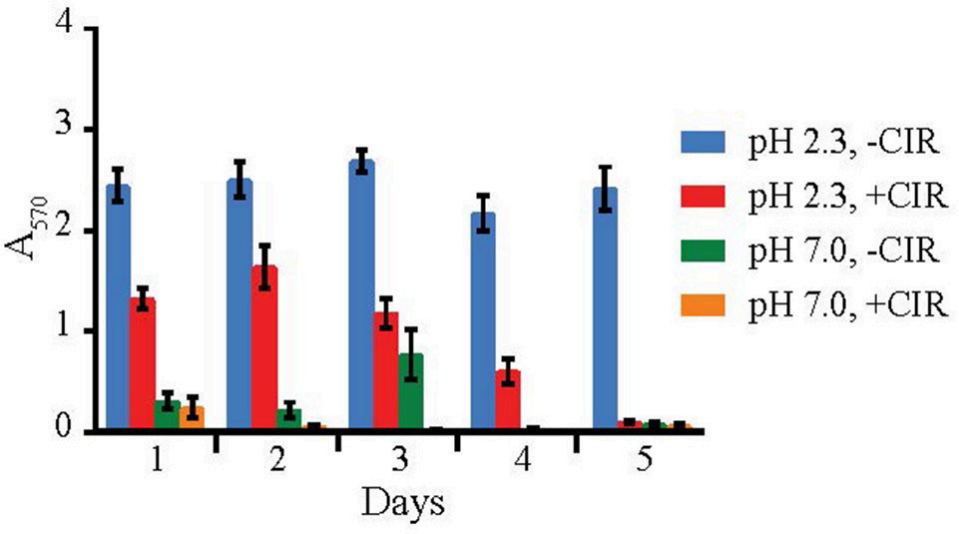
Biofilm formation by *R. taiwanensis* MD1149. Biofilm formation in YPD at pH 2.3 and 7.0 without CIR (-CIR) or under 36 Gy/h (+CIR) was quantified by crystal violet assay.

**Figure 5:**
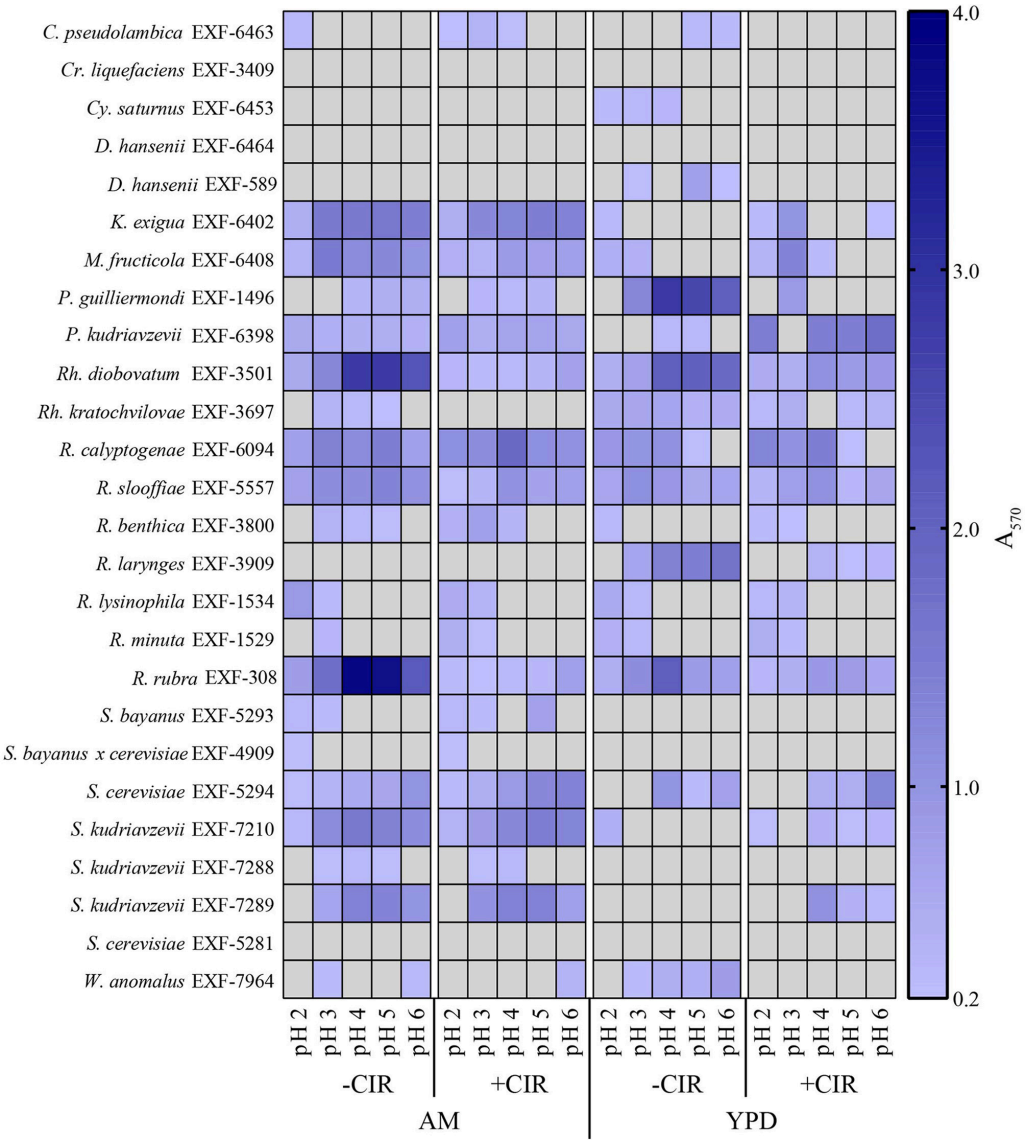
Heat map showing biofilm formation in yeasts. Growth in liquid AM and YPD at pH 2.0, 3.0, 4.0, 5.0, and 6.0. Without CIR and under 36 Gy/h. Biofilms were stained with crystal violet and quantified. Gray area indicates the absence of detectable biofilm, determined by threshold-spectrophotometry at A_570_ < 0.2.

Biofilms are very important in bioremediation, since they offer sorption sites for many divalent cations that are toxic, and thus prevent their migration in the environment. The biofilm-forming capacity in yeasts was estimated with crystal violet assay (O'Toole, 2011) after 24 h incubation (in case of MD1149 it was additionally monitored over 5 days) at pH values 2–6, in the presence and absence of chronic gamma-radiation (36 Gy/h), and in oligotrophic (AM) and rich medium (YPD). This assay was performed on 8 parallels for each strain. The results are summarized in Figures 4, 5. The average absorbance of negative control (no inoculum) was subtracted from each measurement. Difference in A_570_ between the negative control and the sample above 0.2 was interpreted as an indication of biofilm formation.

Out of 27 strains, 3 strains were unable to form biofilms: *Cryptococcus liquefaciens, Debariomyces hansenii*, and *S. cerevisiae* EXF-5281. For the remaining strains, biofilm forming capacity was strongly dependent on the species and physical parameters. For most strains, biofilm formation was inhibited by CIR. However, under specified conditions, biofilm formation in 7 species (*C. pseudolambica, M. fruticola, P*. *kudriavzevii, R. benthica, S. bayanus, S. cerevisiae*, and *S. kudriavzevii*) was moderately enhanced under CIR, based on A_570_ values (Figure 5). In the absence of CIR, pH values below 4 stimulated biofilm formation in 4 yeasts, but inhibited biofilms in the remainder. As pH values decreased to 2.3, in the presence or absence of CIR, MD1149 increasingly formed dense biofilms (Figure 4).

### Organic acid production by MD1149

**Figure 6:**
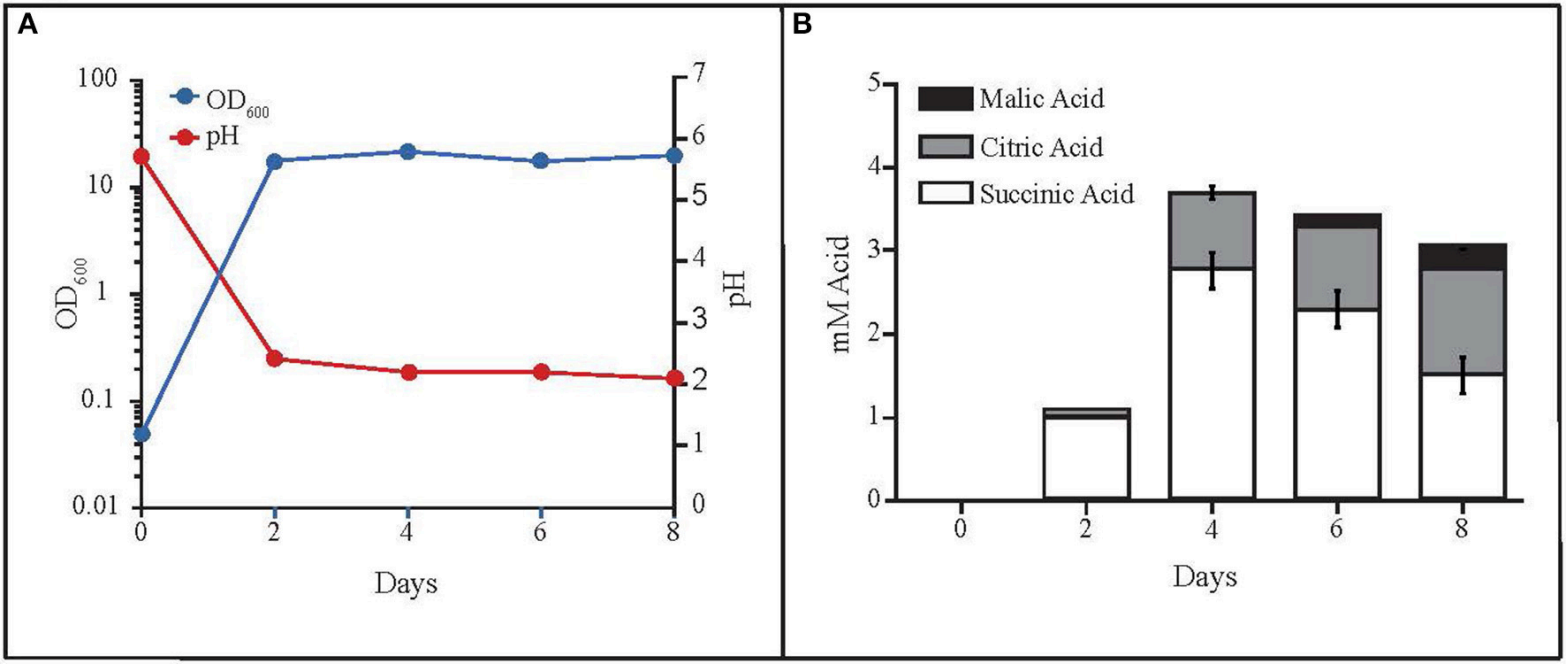
Production of organic acids by *R. taiwanensis* MD1149. **(A)** Growth curve of MD1149 and the pH of the medium over an 8-day time period. **(B)** Quantitation of three organic acids in the SLM (spent liquid medium) for which authentic standards were available (citric, malic, and succinic acids).

While monitoring the growth of MD1149 in YM medium (Figure 6A), we noted unusually high OD_600_ values in stationary phase cultures compared to growth in YPD or AM. This was not due to high cell concentrations in YM, but instead was caused by secretion of metabolites that absorb at 600 nm, which accompanied the drop in pH from 6 to 2.0–2.5 (Figure 6A). These metabolites are now being further investigated.

The rapid pH drop suggested that MD1149 produced significant quantities of organic acids, theoretically in excess of 10 mM depending on pK_a_ values of the acids present. Therefore, we analyzed the SLM by LC-MS to identify any excreted organic acids. We detected the presence of at least six organic acids by LC-MS and LC-MS/MS as well as elemental composition prediction using Waters MassLynx 4.1 software. These acids included citric, homoaconitic, homocitric/homoisocitric (constitutional isomers), malic, rhodotorulic, and succinic. It is noteworthy that organic acids with available reference spectra (citric, malic, and succinic) matched the precursor and product ions from LC-ESI-QTOF (liquid chromatography-electrospray ionization-quadrupole-time of flight-mass spectrometry) spectra published in MassBank (Horai et al., 2010). Furthermore, we procured standards for three of the six organic acids detected (citric, malic, and succinic) and quantitated their abundance (Figure 6B). The combined total concentration of these three acids was ~4 mM, consistent with the idea that these three acids contributed to the decrease in media pH, and that the other identified organic acids (which we were unable to characterize) contribute to the full pH change.

### Sequencing, annotation, and analysis of the MD1149 genome

The estimated size of the genome assembly (without mitochondrial DNA) was 19.58 Mbp, and the final assembly of 181 scaffolds is based on 19.935 Gbp of draft sequence data, which provides 230× coverage of the genome. We identified a total of 26 scaffolds containing either 5′ or 3′ tandem DNA repeats with a sequence TTAGGG, which correspond to the most prevalent telomeric repeats (Teixeira and Gilson, 2005). Based on this result, we can conclude that the genome of MD1149 is organized in at least 13 chromosomes.

**Figure 7:**
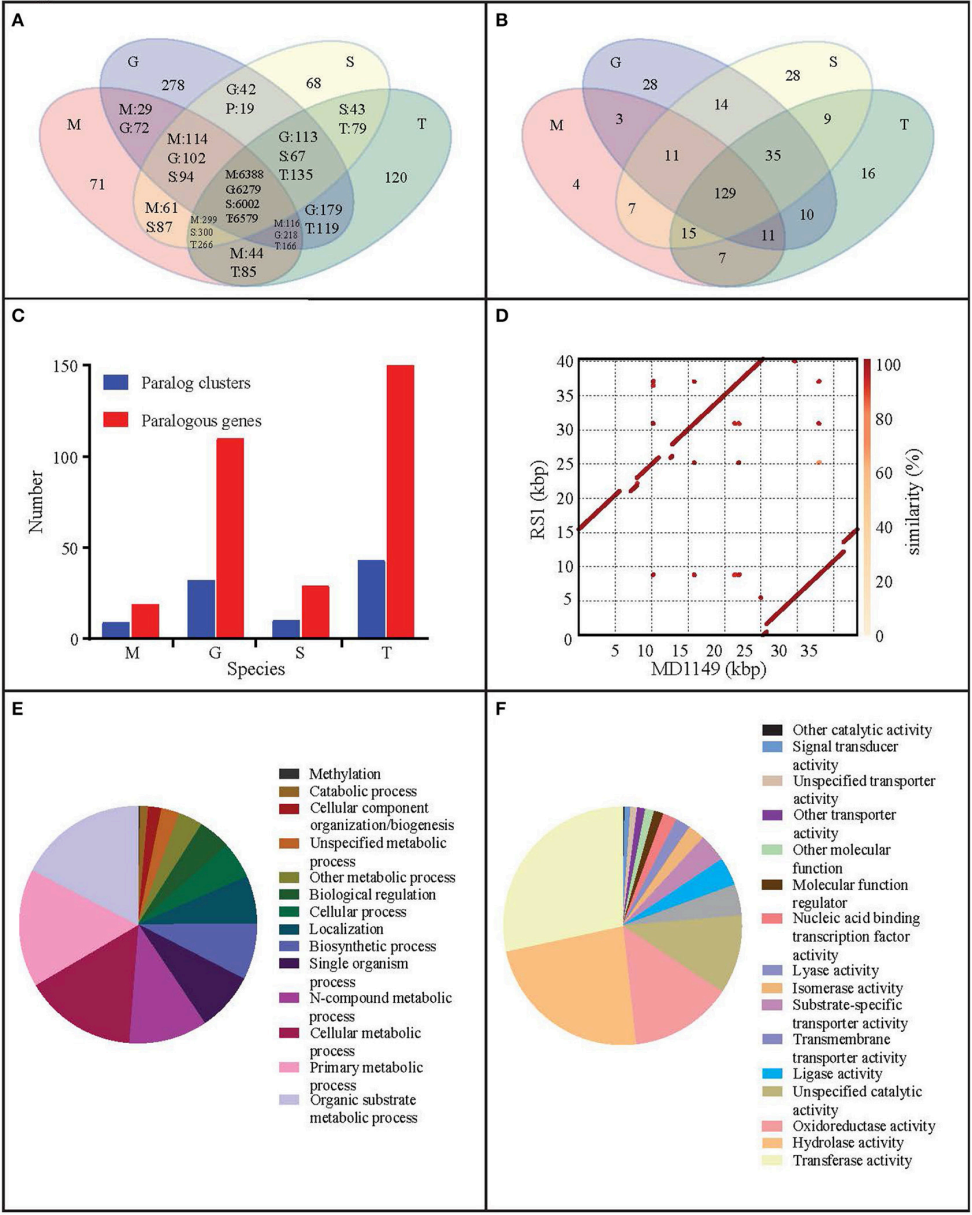
Genome analysis of *R. taiwanensis* MD1149. Venn diagram representation of **(A)** shared/unique genes and **(B)** OrthoMLC groups in *R. taiwanensis* MD1149 (M), *R. graminis* (G), *R*. sp. JG-1b (S), and *R. toruloides* (T). **(C)** Numbers of genes/clusters determined to occur in at least two copies. **(D)** Alignment of mitochondrial DNA of MD1149 and *R. taiwanensis* RS1 (GeneBank: HF558455.1). Percentage of mapped GO annotation translated proteins of MD1149 belonging to two yeast GO-slim functional categories: **(E)** biological process and **(F)** molecular function.

The size of the assembled mitochondrial genome was 38.20 kbp, slightly less than 40.39 kb reported by Zhao et al. (2013a). An alignment of both mitochondrial genomes showed that the sequences were largely syntenic (Figure 7D).

The content of GC pairs was 40.85% in the mitochondrial genome and 61.69% in the nuclear DNA. This finding is comparable to *R. glutinis* (61.87%) and *R. mucilaginosa* (60.54%), but lower than in *R. graminis* (67.76%), which has one of the most GC-rich genomes among available fungal genomes. The number of repetitive sequences was relatively low at 1.49%.

**Figure 8:**
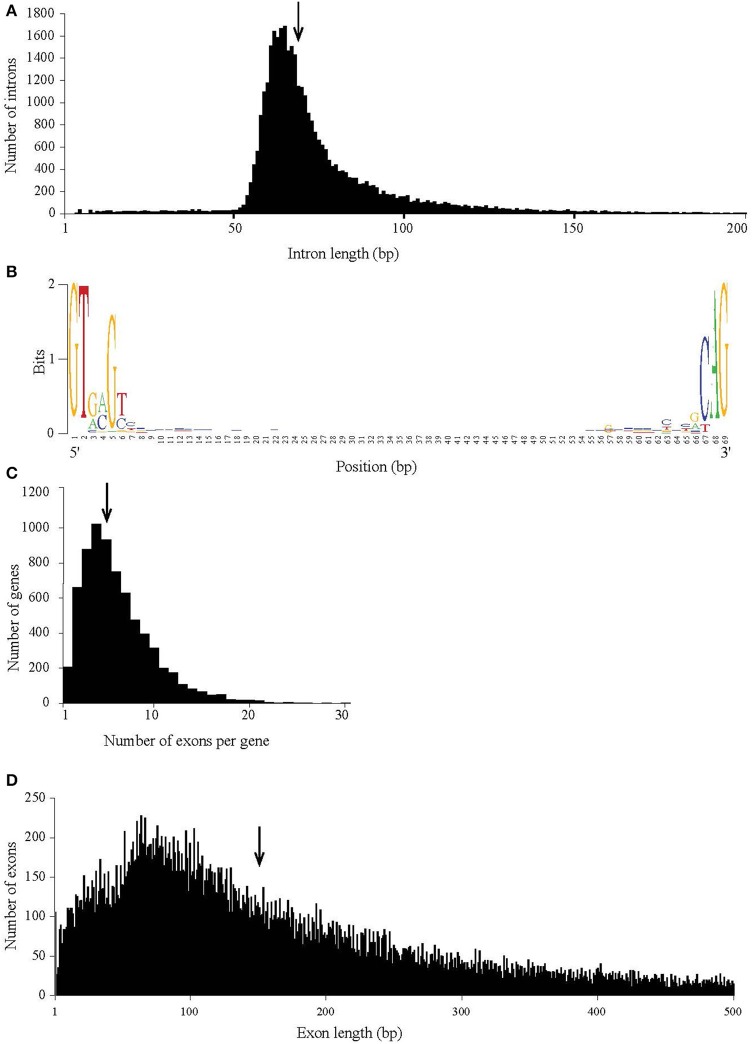
Intron and exon statistics of *R. taiwanensis* MD1149. **(A)** The size distribution of introns. **(B)** The consensus sequence of all median-length introns. **(C)** The distribution of the number of exons per gene. **(D)** The size distribution of exons. The arrow indicates the median.

**Figure 9:**
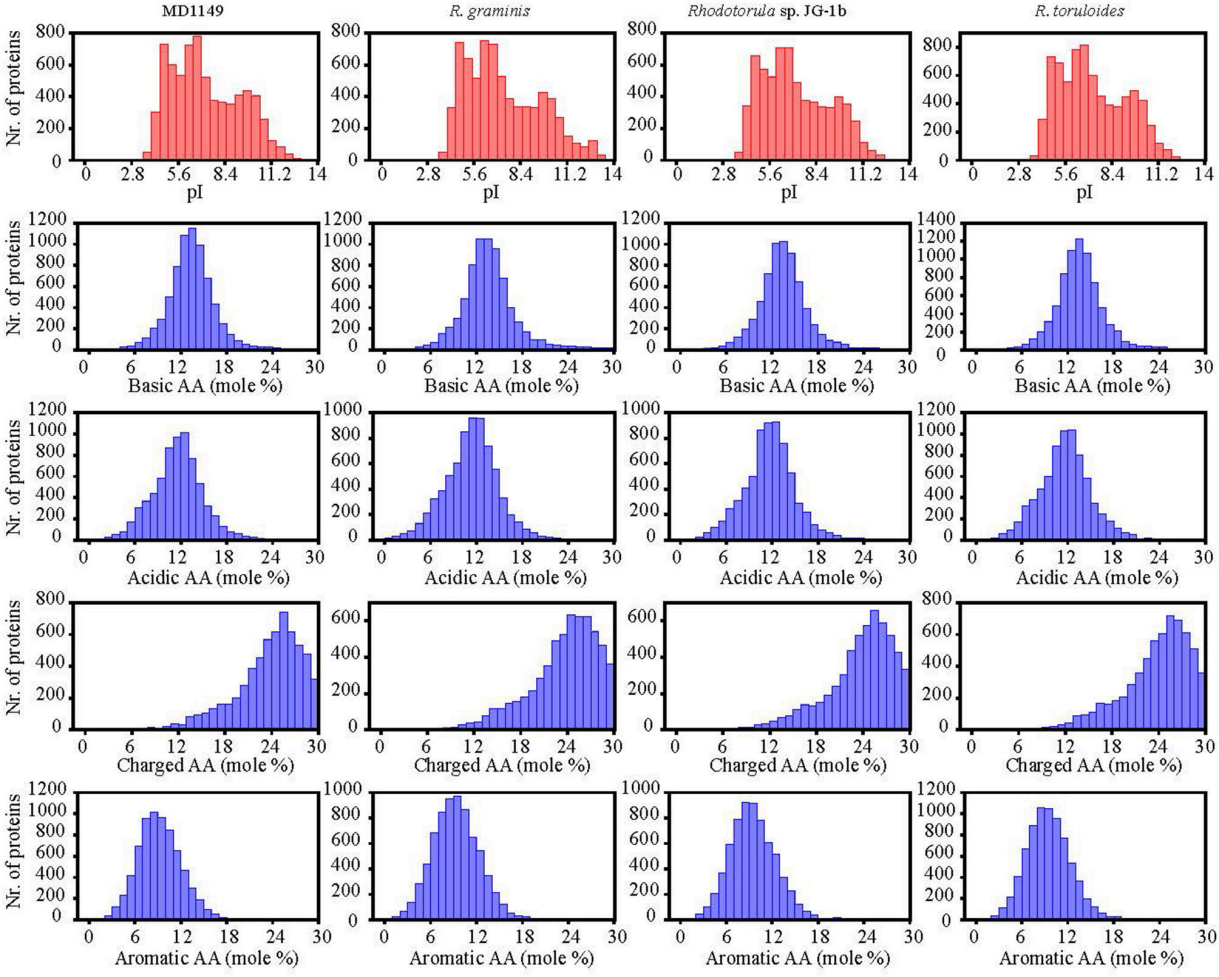
Isoelectric points and amino acid composition of predicted proteins of *R. taiwanensis* MD1149 and related species with sequenced genome.

The number of genes annotated in the genome was 7,122. The genome completeness was estimated by searching the predicted proteome for 1,438 groups of BUSCO. We found 91% complete matches, 7% were fragmented and 2% were missing. More than 97% of MD1149 genes contained introns (Table 2), with an average of 6.2 exons and 5.2 introns per gene (Figure 8C, Table 2) (*R. graminis*: 6.2). The median length of introns was 69 bp (*R. graminis*: 101 bp), and they contained the typical 5′ and 3′ consensus sequences (Figures 8A,B). The median length of the exons was 151 bp (Figure 8D). The average length of the predicted proteins was 531, and their amino acid composition and isoelectric points were comparable to those of other *Rhodotorula* spp. (Figure 9).

**Table 2:** Genome assembly and annotation statistics of *R. taiwanensis* MD1149.

**ASSEMBLY STATISTICS**	
Assembly length (Mbp)	19.58
Mitochondrial genome size (kbp)	38.2
Number of contigs	221
Contig N50	18
Contig L50 (kbp)	345.82
Number of scaffolds	181
Scaffold N50	17
Scaffold L50 (kbp)	388.69
Percentage of scaffolds in gaps	0.15%
Length of repeat-covered regions (bp)	292515
% of assembly covered by repeats	1.49%
GC content	61.69%
Mitochondrial GC content	40.85%
**GENE STATISTICS**	
Number of genes	7122
Gene density (genes per kbp)	0.36
Protein length (amino acids, average)	531
Exon Length (bp, average)	267
Intron length (bp, average)	80
Intron length (bp, median)	69
Number of genes without introns	208
Percentage of genes without introns	2.92%
Exons per gene (average)	6.2
Exons per gene (median)	5
Introns per gene (average)	5.2
GC content of CDS	63.13%
GC content of introns	59.81%
**FUNCTIONAL ANNOTATIONS**	
Genes with KEGG annotation	2774
Genes with Pfam domain	2759
Genes with Transmembrane domain	1249
Genes with SignalP peptide	618

When compared to related species, the distributions of gene families were similar. Only 71 predicted MD1149 proteins and 4 OrthoMLC groups were unique (Figures 7A,B). Although the number of duplicated genes in MD1149 was similar to that in *Rhodotorula* sp. JG-1b, it was much lower than in *R. graminis* and *R. toruloides* (Figure 7C). GO-slim analysis revealed the expected distribution of functional categories (Figures 7E,**F**) for MD1149 genes. To better understand the remarkable radiation resistance of MD1149, we further analyzed the genome for the presence of genes involved in homologous DNA recombination, non-homologous end joining, oxidative stress response, Mn homeostasis, heavy metal resistance, and hydrolases; results are presented (Table 3). The set of genes and their copy number are comparable to other fungi.

**Table 3:** *R. taiwanensis* MD1149 homologs of genes that are known from other fungi to be involved in DNA repair, oxidative stress, Mn homeostasis, resistance to heavy metals, and selected hydrolase genes.

**Representative KEGG genes**	**Function**	**Nr. of homologs in MD1149**	**Homologs numbers (BMF94_)**	
**HOMOLOGOUS DNA RECOMBINATION**				
RAD50	DNA repair protein RAD50	1	4958	
MRE11	Double-strand break repair protein MRE11	1	1623	
RAD57	DNA repair protein RAD57	1, low similarity	1476	
RFA1	Replication factor A1	1	6843	
RAD51	DNA repair protein RAD51	1	4285	
RAD52	DNA repair and recombination protein RAD52	1	1177	
BRCA2	Breast cancer 2 susceptibility protein	1	4266	
RAD54	DNA repair and recombination protein RAD54	2	1178	0432
POLD1	DNA polymerase delta subunit 1	1	4972	
BLM	Bloom syndrome protein, ATP dependent DNA helicase	4	4946	4947
		low similarity	6361	3460
TOP3	DNA topoisomerase III	1	1732	
MUS81	Crossover junction endonuclease MUS81	1	5222	
EME1	Crossover junction endonuclease EME1	0	4418	
**NON-HOMOLOGOUS END-JOINING**				
KU70	ATP-dependent DNA helicase 2 subunit 1	1	5927	
KU80	ATP-dependent DNA helicase 2 subunit 2	1	4455	
RAD50	DNA repair protein RAD50	1	4958	
MRE11	Double-strand break repair protein MRE11	1	1623	
POLL	DNA polymerase lambda	1	4374	
RAD2	Flap endonuclease-1	1	1460	
DNL4	DNA ligase 4	1	5514	
**OXIDATIVE STRESS**				
SOD2 (*S. cerevisiae*)	Fe-Mn family superoxide dismutase	1	4448	
CTA1, CTT1 (*S. cerevisiae*)	Catalase	2	3212	0981
CTT1 (*S. cerevisiae*)	Cytosolic catalase T	2	3212	0981
TSA1, TSA2 (*S. cerevisiae*)	Peroxiredoxin, thioredoxin peroxidase	1	1596	
TSA2 (*S. cerevisiae*)	Stress inducible cytoplasmic thioredoxin peroxidase	1	1596	
PRX1 (*S. cerevisiae*)	Mitochondrial peroxiredoxin, thioredoxin peroxidase	1	1823	
DOT5 (*S. cerevisiae*)	Nuclear thiol peroxidase	1, low similarity to peroxiredoxins	1737	
GPX1, GPX2, GPX3 (*S. cerevisiae*)	Glutathione peroxidase	1	0010	
HYR1 (*S. cerevisiae*)	GPX3 Thiol peroxidase	1	0010	
GTT1 (*S. cerevisiae*)	ER associated glutathione S-transferase	1	4445	
GTO1, ECM4, GTO3 (*S. cerevisiae*)	Omega-class glutathione S-transferase	1	2093	
GRX1, GRX2 (*S. cerevisiae*)	Dithiol glutaredoxin	1	4981	
GRX3, GRX4, GRX5 (*S. cerevisiae*)	Monothiol glutaredoxin	2	1659	3737
GLR1 (*S. cerevisiae*)	Cytosolic and mitochondrial glutathione oxidoreductase	1	0249	
TRX1, TRX2, TRX3 (*S. cerevisiae*)	Thioredoxin	5	6822	6320
			0478	4390
			7059	
TRR2 (*S. cerevisiae*)	Mitochondrial thioredoxin reductase		5997	
NCU05770 (*N. crassa*)	Cytochrome c peroxidase	2	5958	4954
NCU07386 (*N. crassa*)	Fe-Mn family superoxide dismutase	1	2434	
NCU05780 (*N. crassa*)	Theta-class glutathione S-transferase	2	3517	1660
NCU01320 (*N. crassa*)	Microsomal glutathione S-transferase	1	2590	
NCU03339 (*N. crassa*)	Glutathione-disulfide reductase	1	249	
**Mn HOMEOSTASIS**				
SMF2 (*S. cerevisiae*)	Divalent metal ion transporter involved in manganese homeostasis has broad specificity for di-valent and tri-valent metals		0853	
PHO84 (*S. cerevisiae*)	Inorganic phosphate (Pi) transporter, also low-affinity manganese transporter	2	2399	0582
PMR1 (*S. cerevisiae*)	CaMn P-type ATPase transporter	2	5512	2539
BSD2 (*S. cerevisiae*)	Heavy metal ion homeostasis protein	1	5777	
CCC1 (*S. cerevisiae*)	Similar to putative vacuolar FeMn transporter	1	3438	
**RESISTANCE TO HEAVY METALS**				
PCA1	Copper or cadmium transporting P-type ATPase	1	6825	
YCF1	Proteins with high similarity to the yeast vacuolar glutathione S-conjugate transporter with a known role in detoxifying Cd, Hg and As	at least 3	6201	3559
			3360	
COT1, ZRC1	Transporter of heavy metals	1	0535	
**HYDROLASE FAMILIES**				
	GNAT family acetyltransferases	23	5507	6265
			5757	2541
			5786	7019
			4676	7020
			1320	0152
			1591	0305
			4302	0442
			1061	0435
			1055	1739
			1059	3511
			1060	1915
			6163	
	NUDIX hydrolases	15	5675	1605
			5713	3374
			5980	0416
			5976	4813
			1054	1893
			0871	6385
			6480	5468
			4228	
	A/B superfamily hydrolases	25	5552	3298
			1350	3063
			5239	3669
			1589	2387
			6322	4897
			5970	4890
			0382	0636
			0738	0676
			7045	1717
			6437	3427
			6642	3439
			0295	1961
			3113	

**Figure 10:**
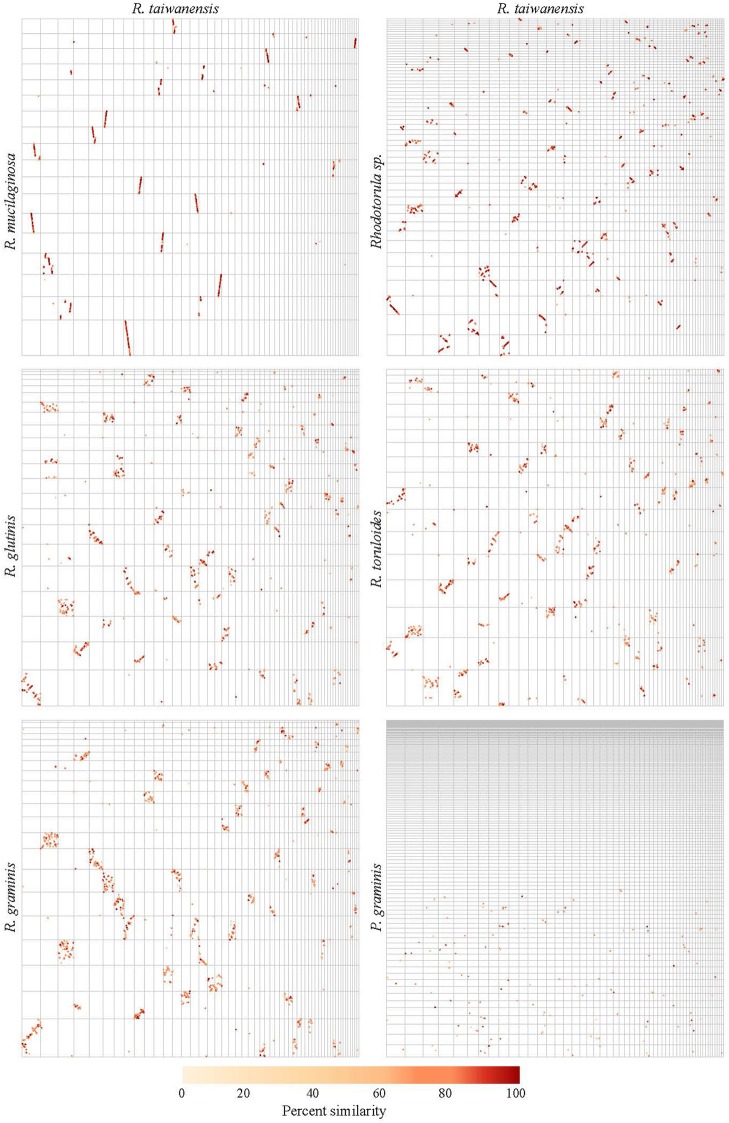
Pairwise genome alignments of *R. taiwanensis* MD1149 and related species. Contigs longer than 100 kbp from the genomes of MD1149 (x-axes) and related species (y-axes) were ordered by length and aligned with Mummer software.

The pairwise genome alignments showed a high level of macrosynteny between MD1149, *R. mucilaginosa* and *Rhodotorula* sp. JG-1b (Figure 10). With *R. toruloides, R. glutinis*, and *R. graminis* the order of the alignable regions was mixed, but the genomic rearrangements appear to have occurred within the same DNA molecules and not between them (Figure 10). This form of evolution is known as mesosynteny, and it was previously thought to be restricted only to filamentous ascomycetes (Hane et al., 2011).

## Discussion

The US Department of Energy (DOE) is the steward of the United States' nuclear waste legacy, comprised of immense volumes of long-lived radioactive environmental waste produced during the Cold War and stored at DOE sites. Over the last six decades, these radioactive wastes have been leaking into the environment, including mixtures of radionuclides, heavy metals and strong acids (e.g., HNO_3_) at levels (e.g., pH < 2.5) that exceed those tolerated by most microorganisms (Brim et al., 2000; Daly, 2000). Despite attempts to neutralize these acidic sites, low pH contamination zones persist, greatly diminishing the prospects for bioremediation at locations close to the originating leaks where the potential benefits are greatest, and where radiation levels are highest (Daly, 2000; Shelobolina et al., 2003).

We studied 16 ascomycetous and 11 basidiomycetous yeasts isolated from diverse environments including arctic ice, acid mine drainage, red wine, and apple juice, as well as dry environments with elevated temperatures (Table 1). Whereas many yeasts and filamentous fungi are reported to be resistant to various extreme environments, there are no reports of yeasts being resistant to high-level CIR. All 27 yeasts were able to survive an acute exposure to gamma-rays over the range 0.3–3.2 kGy: 8 extremely resistant yeasts displayed D_10_ values between 2.0 and 3.2 kGy; 14 yeasts were moderately resistant with D_10_ values between 1–2 kGy; and 5 yeasts were relatively sensitive, with D_10_ values as low as 300 Gy, but still more resistant than many bacteria (Daly, 2012). For comparison, the D_10_ of the soil bacterium *Shewanella oneidensis* is 70 Gy (Daly, 2012). Thus, this survey elevates yeast to the frontier of biology's most radiation-resistant representatives (Daly, 2012). In the context of bioremediation of DOE sites, CIR resistance is most relevant: 18/27 strains were able to grow under 36 Gy/h at pH 2.3, comparable to dose rates and pH values reported for sediments beneath Hanford tank SX-108 (Fredrickson et al., 2004). Surprisingly, among the surveyed yeasts, we show that chronic and acute radiation responses are not always aligned: *S. cerevisiae* strain EXF-5294 (D_10_, 3.2 kGy) did not grow under 36 Gy/h, and similarly for *S. kudriavzevii* EXF-7288 (D_10_, 1.5 kGy). A special focus is placed on *R. taiwanensis* MD1149, isolated from an acid mine drainage facility. MD1149 is capable of growth under 66 Gy/h at pH 2.3 (Figure 1C).

The concentration of contaminant heavy metals at DOE sediments can reach 10–30 μM (Fredrickson et al., 2004). Many microorganisms are reported to resist the toxic effects of metals by immobilizing and/or transforming those metals to less toxic chemical states (Brim et al., 2000; Fredrickson et al., 2000). Far fewer microorganisms are known to be able to transform metals at low pH, and there have been no published reports on any organism capable of transforming metals at low pH under high-level CIR. We ranked the 27 yeasts for their resistance to two heavy metals that predominate at DOE waste sites: 1. ionic Hg^2+^ in the form of HgCl_2_, and Hg as an organo-Hg compound merbromin; and 2. chromium in the form of CrCl_3_ (Cr^3+^) and K_2_Cr_2_O_7_ (Cr^6+^), as presented in Table 1. Redox-active heavy metals propagate ROS in cells and typically are more toxic than their covalently-bound counterparts. Consistently, we show Hg^2+^ and Cr^6+^ were the most toxic, followed by Cr^3+^, then merbromin. The ability of many of the tested yeasts to grow in the presence of 50–100 μM concentrations of Hg or Cr thus elevates these radiation-resistant simple eukaryotes to the forefront of metal resistances encountered in the natural world: 14 of the strains were able to grow in the presence of 25 μM HgCl_2_; 2 strains, MD1149 and *R. kratochvilovae*, grew in 50 μM HgCl_2_; and 14 strains grew in 1 mM merbromin (Table 1). Unlike Hg^0^ and Hg^2+^, redox-active Cr can cycle between several oxidation states between +2 to +6, with the most stable forms in the environment being hexavalent Cr^6+^ and trivalent Cr^3+^. These oxidation states have different chemical properties. For example, Cr^3+^ is relatively insoluble in the environment and is far less toxic than Cr^6+^, which is highly soluble and generates ROS in cells (Viti et al., 2014). Indeed, most yeasts were able to grow at concentrations of 500 μM merbromin or CrCl_3_; and three species, *W. anomalus, Cyberlindnera saturnus* and *C. pseudolambica* grew at even higher concentrations of these heavy metals (Table 1).

In bioremediation, biofilm formation is a highly desirable characteristic because the polysaccharide/protein extracellular matrix can bind/adsorb cations and reduce their migration in the environment. In the past few decades, most research on biofilms has focused on medically important bacteria and a few yeasts (Niemira and Solomon, 2005). Importantly, we report that biofilm formation in some yeasts is facilitated by chronic gamma radiation (Figure 5). In particular, MD1149 is capable of forming biofilms and growing in the presence of heavy metals under 36 Gy/h (Figures 3, 4). We also show that MD1149 produces abundant carboxylic acids (e.g., succinic acid) (Figure 6), similarly to *Rhodotorula glutinis* (Glass and Bhattacharjee, 1971), which is expected to facilitate metal transformation and metal accumulation in biofilms formed at low pH under CIR, but more evidence is needed.

The yeast we judged most suitable for bioremediation of acidic radioactive DOE waste sites was MD1149. To further develop this basidiomycete as a bioremediating platform, we subjected MD1149 to whole genome sequencing, then compared the genome to three other *Rhodotorula* species (*R. graminis, Rhodotorula* sp. JG-1b, *R. toruloides*). The complete sequence of the MD1149 genome is organized into at least 13 chromosomes (Figure 1F). The sequence-based features are summarized (Table 2), and when compared to the other *Rhodotorula* spp., the genome is unremarkable with respect to its size and GC content. Moreover, compared to other basidiomycetes the genome and the predicted proteome are relatively small (Mohanta and Bae, 2015). Viewed from the perspective of radiation resistance, the MD1149 genome and the predicted proteome exemplify characteristics found in many other sequenced species across the tree of life (Paul et al., 2014; Deligios et al., 2015; Goordial et al., 2016; Zhang et al., 2016; Matrosova et al., 2017), *viz*. The predicted DSB homologous recombination and non-homologous end-joining repair functions of MD1149, as well as its enzymatic antioxidant enzymes, are unremarkable (Table 3). Further, MD1149 encodes numerous genes commonly implicated in generating low molecular weight (LMW) metabolites (e.g., orthophosphate). They include acetyltransferases of the GNAT family, Nudix hydrolases, a/b superfamily hydrolases and calcineurin family phosphoesterases, which are present in many fungi (Zhao et al., 2013b). For most of these predicted hydrolases, and phosphatases in particular, their substrate specificities are either unknown or the affinity of known substrates is extremely low. It is likely that these predicted MD1149 enzymes, similar to *D. radiodurans*, participate in the degradation of nucleic acids, proteins and lipids (Makarova et al., 2001). The prediction of so many hydrolase functions in MD1149, which also encodes systems for Mn accumulation (Table 3), is expected to give rise to high intracellular concentrations of low molecular weight Mn^2+^ antioxidants. The hydrolase genes may therefore play a role in MD1149's extreme radiation resistance, yielding high intracellular concentrations of the organic and inorganic ligand-precursors of Mn^2+^ antioxidants that maintain proteome functionality under oxidative stress (Daly et al., 2007, 2010; Sharma et al., 2017).

Physicochemical cleanup technologies that could be used to decontaminate the immense volumes of soils, sediments and groundwaters at DOE facilities are prohibitively expensive and dangerous. Thus, the use of microorganisms to stabilize and/or detoxify such waste environments may be a viable alternative (Prakash et al., 2013). A bioremediation strategy based on the basidiomycete MD1149 and other yeasts (this study; Chandran and Das, 2012) now offers a more promising path to stabilization of DOE sites than *Deinococcus* spp., which are intolerant of low pH and heavy metals. Remarkably, MD1149 is highly resistant to Hg, Cr and CIR, capable of forming biofilms under 36 Gy/h at pH 2.3, and surviving acute doses of 2.5 kGy at pH 2.3. Importantly, it is reported that *Rhodotorula* spp. are genetically tractable (Takahashi et al., 2014), and we anticipate that MD1149 could be a good candidate for fungal-based CRISPR/Cas9 technologies (DiCarlo et al., 2013). Thus, the proposed use of MD1149 and other fungi for treatment of environments where radiation, low pH, and heavy metals are the principle factors limiting microbial survival and function appears to be a realistic approach given these early data.

## Author contributions

RT, MD, and LD: designed research; RT, VM, OG, RV, PK, EG, CZ, BS, ML, SM, BR, JS, CD, TH, KF, and NG-C: performed research; RT, CG, SM, MC, and CZ: analyzed data; and RT, LD, and MD: wrote the paper.

### Conflict of interest statement

The authors declare that the research was conducted in the absence of any commercial or financial relationships that could be construed as a potential conflict of interest.

## Footnotes

^1^http://www.ex-genebank.com

^2^http://www.bd.com/europe/regulatory/Assets/IFU/Difco_BBL/242820.pdf

^3^https://github.com/carolzhou/genome
